# *Drosophila melanogaster* Models of Metal-Related Human Diseases and Metal Toxicity

**DOI:** 10.3390/ijms18071456

**Published:** 2017-07-06

**Authors:** Pablo Calap-Quintana, Javier González-Fernández, Noelia Sebastiá-Ortega, José Vicente Llorens, María Dolores Moltó

**Affiliations:** 1 Department of Genetics, University of Valencia, Campus of Burjassot, 46100 Valencia, Spain; pablo.calap@uv.es (P.C.-Q.); javier.gonzalez-fernandez@uv.es (J.G.-F.); noelia.sebastia@uv.es (N.S.-O.); dolores.molto@uv.es (M.D.M.); 2 Biomedical Research Institute INCLIVA, 46010 Valencia, Spain; 3 Centro de Investigación Biomédica en Red de Salud Mental CIBERSAM, Spain

**Keywords:** *Drosophila*, metal homeostasis, iron, copper, zinc, frataxin, ATP7, dZip99C, neurodegeneration, heavy metal toxicity

## Abstract

Iron, copper and zinc are transition metals essential for life because they are required in a multitude of biological processes. Organisms have evolved to acquire metals from nutrition and to maintain adequate levels of each metal to avoid damaging effects associated with its deficiency, excess or misplacement. Interestingly, the main components of metal homeostatic pathways are conserved, with many orthologues of the human metal-related genes having been identified and characterized in *Drosophila melanogaster*. *Drosophila* has gained appreciation as a useful model for studying human diseases, including those caused by mutations in pathways controlling cellular metal homeostasis. Flies have many advantages in the laboratory, such as a short life cycle, easy handling and inexpensive maintenance. Furthermore, they can be raised in a large number. In addition, flies are greatly appreciated because they offer a considerable number of genetic tools to address some of the unresolved questions concerning disease pathology, which in turn could contribute to our understanding of the metal metabolism and homeostasis. This review recapitulates the metabolism of the principal transition metals, namely iron, zinc and copper, in *Drosophila* and the utility of this organism as an experimental model to explore the role of metal dyshomeostasis in different human diseases. Finally, a summary of the contribution of *Drosophila* as a model for testing metal toxicity is provided.

## 1. Introduction 

Model organisms have been extensively used to unravel basic and conserved biological processes. The fruit fly, *Drosophila melanogaster* (hereinafter *Drosophila*), is one of the most studied eukaryotic organisms and has made fundamental contributions to different areas of biology. *Drosophila* has also gained appreciation as a useful model organism of human diseases. Comparative genomic studies estimate that up to 75% of the human genes implicated in diseases are conserved in *Drosophila* [1]. The similarity between human and *Drosophila* genomes is not limited only to genetic elements, but also to the relationship between them, with numerous examples of conserved biological mechanisms. The *Drosophila* genome is smaller in size and has a smaller number of genes compared to the human genome, which facilitates genetic studies [2]. Accordingly, many human gene families are composed of paralogues with redundancy of overlapping functions that correspond to a single gene or a smaller gene family in *Drosophila*.

Flies are greatly appreciated in the laboratory because they are undemanding animals, with a short generation interval and inexpensive maintenance. Flies can easily be handled, bred and genetically manipulated in large numbers. Currently, there is a high number of genetic tools available in *Drosophila* that allows researchers to address some of the outstanding questions concerning basic processes underlying human diseases. Furthermore, fly models can be exploited very successfully to discover the genetic modifiers of disease phenotypes by means of modifier screens [3]. This methodology is central in identifying novel genes that function in the same disease processes and to progress in understanding the disease pathology. In turn, this is essential for researching appropriate therapies. Nowadays, different human diseases have been modeled in *Drosophila*, which cover a wide range of physiological alterations, including metabolic dysfunctions and neurodegeneration [4].

*Drosophila* is also useful for studying potential toxic effects of different compounds, such as metals [5]. As flies have a short biological life cycle, it is possible to address metal toxicity during development and adulthood. Survival, neuronal function and behavior assays are easy to perform in this organism. It is also simple to search for toxicity-mediated mechanisms at the molecular level in *Drosophila* [6,7].

This review focuses first on the conserved components that control metal homeostasis in flies. Following this, it provides instructive examples about what we have learned from *Drosophila* as a model for inherited diseases related to metal dysregulation and as a model for testing metal toxicity.

## 2. *Drosophila* Genes Involved in Metal Homeostasis

Approximately 25% of proteins require metals to perform their function [8]. Iron (Fe), copper (Cu) and zinc (Zn) are essential transition metals that organisms assimilate from food. Homeostatic control of these metals at systemic and cellular levels is vitally fine-tuned to ensure their availability as cellular nutrients. This fine-tuning also prevents detrimental effects of their deficiency, excess or misplacement. Proteins for import, transport, storage, excretion and regulation of metals are essential components of pathways for metal metabolism and homeostasis. Interestingly, the main players of these pathways are conserved through evolution, with many orthologues of the human metal-related genes having been identified and characterized in *Drosophila*. Here, we provide an overview of the uptake, transport and efflux of Fe, Cu and Zn at the cellular level in flies.

### 2.1. Iron

Iron (Fe) is required for the survival of almost all organisms, because it plays a crucial role in many biological processes, such as oxygen transport, cellular respiration, gene regulation and DNA biosynthesis. Fe participates in such processes through the heme and iron-sulfur cluster (ISC) prosthetic groups in which Fe shows its ability to exchange electrons with different substrates.

Fe deficit compromises the function of the metabolic pathways in which Fe acts as an important cofactor. A misplaced and increased level of Fe promotes the formation of reactive oxygen species (ROS) through the Fenton–Haber–Weiss reaction [9], including the highly reactive hydroxyl radicals. This results in glutathione consumption, lipid peroxidation and DNA damage, which finally can compromise cell viability.

**Figure 1:**
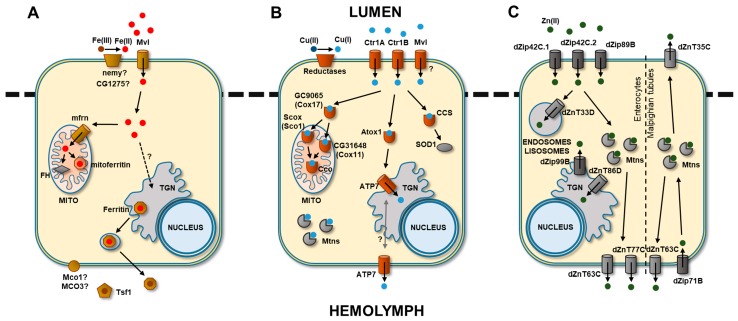
Main pathways of (**A**) iron, (**B**) copper and (**C**) zinc uptake, storage and export in *Drosophila melanogaster*. TGN, trans-Golgi network; MITO, mitochondria. Question marks represent an unknown mechanism. Arrows represent the direction of the metal transport.

*Drosophila* shares many key genes involved in iron metabolism with mammals (Table 1, Figure 1A). *Malvolio* (*Mvl*), the *Drosophila* orthologue of the mammalian *Divalent metal transporter-1* (*DMT1*) [10] is expressed in the fly midgut and has been proposed to function as an importer for dietary iron in a similar way to its mammalian counterpart [11]. Mammals and insects store iron absorbed in a bioavailable form of ferrihydrite inside ferritin, a protein formed of heavy and light chain subunits (coded by the *Fer1HCH* and *Fer2LCH* genes respectively in *Drosophila*). While ferritin is mainly a cytosolic protein in mammals, it is found in the secretory system (endoplasmic reticulum (ER), Golgi apparatus and secretory vesicles) in most insects, including *Drosophila*, and is secreted in the fly hemolymph in large amounts [12,13]. Transferrin 1 is also an iron-binding protein abundant in the hemolymph for which multiple functions have been suggested in insects. Nevertheless, it is still unsolved whether this protein serves as an iron transport carrier between cells in a similar manner to the mammalian transferring [14,15]. Iron absorption and metabolism are post-transcriptional processes regulated by the IRP/IRE system, which is conserved through diverse taxonomic groups [12,16]. When Fe concentration is low, iron regulatory proteins (IRPs) bind to iron-responsive elements (IREs) on the 5′UTR and 3′UTR mRNAs of their target proteins, which controls translation initiation or mRNA stability (reviewed in [17]). Two IRP genes (*Irp-1A* and *Irp-1B*) have been described in *Drosophila* and have both shown aconitase activity, but only Irp-1A functions as an iron regulatory protein [18].

Genes involved in mitochondrial Fe metabolism are also conserved between flies and humans. A mitochondrial ferritin has been identified in both organisms (*Fer3HCH* is the encoding gene in *Drosophila*), which has shown an important antioxidant role through the regulation of mitochondrial Fe availability [19,24]. In fact, the expression of human mitochondrial ferritin in frataxin-deficient cells protects mitochondria from oxidative stress, which is a situation that provokes mitochondrial iron overload [25,26]. Similarly, the expression of *Fer3HCH* in frataxin-deficient flies is able to extend the mean lifespan of the adult individuals [27]. Bridwell-Rabb et al. propose that frataxin is an iron chaperone involved in the regulation of ISC biosynthesis [28].

*Drosophila* also has other orthologues with known iron-related functions in mammals that remain to be fully elucidated in flies. The *Duodenal cytochrome b* (*Dcytb*) is highly expressed in the intestinal epithelia of mammals and has ferric reductase activity to convert the inorganic dietary iron(III) state, Fe^3+^ to the iron(II) state and Fe^2+^ in the absorption process. In *Drosophila*, two candidates for this function have been proposed, which are *no extended memory* (*nemy*) and the gene with the annotation ID of *CG1275* [21]. Fe^2+^ is oxidized to Fe^3+^ for efflux from intestinal enterocytes into the circulatory system. Mammalian hephaestin, a transmembrane copper-dependent ferroxidase, plays this role and mediates iron efflux in cooperation with the basolateral iron transporter, ferroportin 1. Another copper-dependent oxidase enzyme, ceruloplasmin, participates in the iron transport in plasma in association with transferrin, which carries iron in the ferric state. It has been suggested that the *multicopper oxidase-1* (*Mco1*) and the *multicopper oxidase-3* (*MCO3*) may be involved in iron homeostasis in *Drosophila* showing hephaestin-like [22] and ceruloplasmin-like [29] functions, respectively. It has been recently reported that *Mco1* orthologues from diverse insect species, including *Drosophila*, function as ascorbate oxidases [23], and there is accumulating evidence strongly suggesting that ascorbate within mammalian systems can regulate cellular iron uptake and metabolism [30]. Thus, iron homeostasis might also be influenced by the ascorbate redox state in *Drosophila*.

Finally, there are some mammalian genes participating in Fe metabolism with no known orthologues in flies, such as those genes encoding the transferrin receptor, the iron export protein, ferroportin or the iron hormone, hepcidin [31]. It may account for differences in the control of systemic Fe levels between insects and mammals. However, *Drosophila* has become a powerful model for studying human disorders caused by mutations in the main components of cellular Fe homeostasis pathways. Friedreich’s ataxia is an example of these human conditions, and we report in this review the contribution of *Drosophila* as an experimental model in the advancement of knowledge in this disease.

### 2.2. Copper

Copper (Cu) is a vital co-factor for enzymes involved in a wide range of roles, from oxidative stress defense (superoxide dismutase (SOD)) to pigmentation (tyrosinase) or energy production (cytochrome c oxidase). Its usefulness for life comes from its ability to change its oxidation state between the cupric (Cu(II)) and cuprous (Cu(I)) forms.

In mammals, copper is distributed throughout the organism in two steps [32]. First, Cu is absorbed in the apical surface of the enterocytes of the small intestine to be exported to the circulation bound to serum proteins. Following this, Cu is absorbed by the liver, where it is bound to the serum ceruloplasmin and exported again to the circulation for its use by the peripheral tissues. The liver is also responsible for the removal of excess Cu, exporting it to the bile for its elimination through the feces. The main transporter for cellular import of Cu is CTR1 (copper transporter 1), which belongs to a family of small proteins with three transmembrane domains [33]. Before Cu is transported by CTR1, Cu(II) is reduced to Cu(I) by reductases in the plasma membrane. The transporter DMT1, which is involved in iron uptake and reabsorption, could also import Cu, but this possibility requires further research [34,35].

In *Drosophila*, copper is transported in a single step from the midgut to all tissues through the circulation. The multicopper oxidase MCO3 is similar to ceruloplasmin in its ferroxidase activity [29], but it has not been detected in the hemolymph [36,37]. Some authors suggest that another MCO orthologue could still be implicated in Cu homeostasis [38]. As for Cu excretion, it has been observed that in larvae growing in media with a high Cu concentration, the metal accumulates in the Malpighian tubules [39]. These organs are located between the midgut and the hindgut, which regulate fluid and ion balance [40]. Malpighian tubules may absorb excess Cu for its excretion to the hindgut and elimination through the feces [38]. There are three *CTR1* genes in *Drosophila* (Table 2) with some differences in their expression patterns [41]. *Ctr1A* is ubiquitously expressed and is required for survival [42], suggesting that it is the primary transporter for dietary Cu uptake. *Ctr1B* expression in the gut is transcriptionally induced when there is a Cu limitation and repressed in the case of excess [41]. The expression of *Ctr1C* seems to take place mainly in the male germline. In *Drosophila*, *Mvl* (the orthologue of the human *DMT1*) could also contribute to Cu uptake, since a reduction in *Mvl* activity causes an increased sensitivity to the Cu limitation in females and S2 cells [43].

Once copper has entered the cell (Figure 1B), it is transported to its target proteins by Cu-specific chaperones with a high affinity for the metal: CCS, COX17 and ATOX1. The CCS (copper chaperones for superoxide dismutase) provide Cu for SOD1 [44]. COX17 transfers Cu to the proteins, COX11 and SCO1, which are located in the inner mitochondrial membrane and deliver the metal to the cytochrome c oxidase [46]. In mammals, ATOX1 transfers Cu to ATP7A and ATP7B, which are two P-Type ATPase transporters. ATP7A donates Cu to different enzymes in the secretory pathway [33,49,50]. It is also required for Cu transport across the blood brain barrier (BBB) and from intestinal enterocytes into the circulation. ATP7B delivers Cu to ceruloplasmin, and in the case of Cu excess, Cu moves to the biliary canalicular membrane for its excretion to the bile duct and its elimination through the digestive tract [49,50,56]. One important characteristic of both transporters is their capacity for trafficking between the trans-Golgi network and the cell membrane in response to Cu levels.

In *Drosophila*, orthologues of Atox1, CCS, SCO1 and COX17 have been found, and they are thought to perform the same roles as in mammals [45,47,48]. However, in the fly, there is only one Cu transporting P-type ATPase named ATP7, which is expressed in all tissues [51,52]. This transporter is similar to ATP7A, since it is required for transferring Cu to enzymes in the secretory pathway [52,53,54] and from the gut cells to the circulatory system [55]. Moreover, the similarity between ATP7 and ATP7A has also been observed in functional assays and in the domains that share both proteins [51]. Despite the fact that the efflux activity of ATP7 has been proven [53,55], the translocation of the transporter from the trans-Golgi network to the cell membrane in response to the Cu levels has not been demonstrated yet [38].

Copper levels must be tightly controlled because besides being critical for life, Cu can also be potentially toxic. It can catalyze the formation of hydroxyl radicals through Fenton reactions and alter the structure of proteins [57,58]. As a mechanism of scavenging and/or storage of Cu and other metals, all eukaryotes express a group of proteins known as metallothioneins. These are low weight proteins with a high content of cysteines that have a great metal binding capacity [59,60,61]. Metallothioneins are expressed at a basal level and are strongly induced in response to heavy metal load in order to cope with their toxicity [59,60,61]. In mammals, there are four major members of this protein family, namely MT-I, MT-II, MT-III and MT-IV [62]. In *Drosophila*, five different metallothioneins have been described, namely MtnA, MtnB, MtnC, MtnD and MtnE [63,64].

The transcription factor MTF-1 (metal-responsive transcription factor-1) is the main regulator of the response to heavy metal load, including Cu [61,65,66]. In normal conditions, the majority of MTF-1 is located in the cytoplasm. In conditions of heavy metal load, MTF-1 accumulates in the nucleus and binds to the metal response elements (MRE) to induce the expression of its target genes, which include the metallothioneins, both in mammals and *Drosophila* [66,67]. In *Drosophila*, MTF-1 is also responsible for the induction of *Ctr1B* expression under limited Cu [68] and *ATP7* in response to excess Cu [55], while regulation of *CTR1*, *ATP7A* or *ATP7B* by MTF-1 has not been observed in mammals [38].

### 2.3. Zinc

Zinc (Zn) is an indispensable and ubiquitous trace element present in many tissues in a wide range of organisms and is involved in many general cellular functions. It has multitudinous effects on growth, development, immune system and nervous system function [69]. As a regulatory, catalytic and structural component, Zn is also needed for proteins involved in DNA repair, transcription, translation and protein signaling, which requires tight homeostasis. It modulates the stability of cell membranes by reducing peroxidative damage [70] and provides protection against the disruption of cells [71] by maintaining the structure and function of the membrane barrier [72]. The majority of Zn is bound with high affinity to metalloproteins and subsequently is said to be non-bioavailable [73]. Furthermore, approximately 40% of putative zinc-binding proteins are transcription factors carrying zinc-binding domains, such as zinc finger motifs, zinc cluster and zinc twist. These enable specific gene regulatory processes to be activated [73]. Other studies have reported the ability of Zn to act as a neurotransmitter and a second messenger [74].

The bioavailable Zn concentration is maintained at a nanomolar level through uptake, storage and secretion [75]. Several studies have indicated that Zn transporters are likely to be the main mechanism of Zn movement in cells, although diffusion of Zn ions may occur under physiological conditions [76]. In mammals, there are two Zn transporter families. One is composed of solute-linked carrier genes, namely the SLC39a/Zrt-Irt-like protein family (also known as ZIP proteins), while the other family includes cation diffusion facilitator genes, the SLC30a family (also known as ZnT proteins) [77].

The ZnT family of transporters specifically promotes the transport of Zn from the cytosol to extracellular environment or transfers Zn into organelle compartments within the cell, thereby reducing the concentration of cytosolic zinc. The distribution of ZnTs is widespread, although some are cell-specific [78]. The ZIP family is comprised of genes that are ubiquitously expressed in all organisms. This family promotes the transport of Zn from the extracellular environment into the cytoplasm or from the cellular organelles to the cytoplasm [79]. Thus far, at least fourteen ZIPs and ten ZnTs [77] have been identified in mammals, while ten ZIPs and seven ZnTs orthologues have been identified in *Drosophila* that control the zinc homeostasis (Table 3).

Catsup (*Catecholamines up*) and foi (*fear of intimacy*) are the best characterized Zn transporters in *Drosophila*. *Catsup* is the orthologue of the human *hZIP7* gene. The encoded protein is localized in the ER and Golgi apparatus, playing a more general role in the maintenance of the function of these organelles. Catsup is present in a punctate pattern in the cell body, axonal and dendritic synapses of neurons in the central brain in *Drosophila*. It has been shown to play a role as a negative regulator of the catecholamine biosynthesis pathway [112], as well as in synaptic transport and release of dopamine [113]. Catsup mutations produce abnormal accumulation of membrane proteins, such as Notch receptor, EGFR and APPL in ER and Golgi apparatus [114].

*Foi* has been suggested to be the orthologue of the human genes *hZIP6* and *hZIP10*, with the encoded protein located in the basolateral cell membrane. It is implicated in cell migration in the gonad and trachea morphogenesis [115,116], as well as in embryonic glial cell patterning [117]. Foi is also necessary for correct specification and consequent differentiation of mesodermal derivatives, acting as a Zn transporter that regulates Zn cellular homeostasis. Its function can be partially replaced by other Zn transporters of the same family that have a similar subcellular localization [118].

In *Drosophila*, *dZip42C*.1, *dZip42C*.2 and *dZip89B* (likely orthologues of *hZIP1*, *hZIP2* and *hZIP3*) are the most important transporters for absorption of Zn from the intestinal lumen. All of them are located in the apical membrane of enterocytes (Figure 1C), and their expression can be affected by dietary Zn levels (*dZip42C.1* and *dZip42C*.2) [119] or can be independent of Zn content acting as a constitutive Zn transporter with a lower affinity for this metal (*dZip89B*) [120].

The Zn release to the hemolymph is mediated through two overlapping Zn exporters, *dZnT63C* and *dZnT77C* (orthologues of *hZnT1* and *hZnt10*) both located in the basolateral membrane of enterocytes. Ubiquitous RNA interference (RNAi) of *dZnT63C* results in developmental arrest at the larval stage, lethality before pupae formation and Zn accumulation under physiological conditions [121]. Knocking down *dZnT77C* also produces a greater sensitivity to Zn deficiency [119], demonstrating that they are key Zn transporters in dietary Zn absorption.

Intracellular Zn homeostasis and transport is poorly understood in *Drosophila*. Even so, *dZnT86D* is expressed in Malpighian tubules, midgut and other organs. The encoded protein is located in the Golgi apparatus [122] and has been shown to be important for the control of Zn levels. Ubiquitous knockdown of *dZnT86D* causes a severe reduction in Zn level in the whole fly, while gut-specific knockdown of this gene produces little effect on Zn metabolism, but local alteration of the Zn level in Golgi apparatus. The larval lethality resulting from ubiquitous silencing of *dZnT86D* is partially rescued by expression of *hZnT7*, which corroborates that it is the orthologue [119]. 

*dZnT33D* is another intracellular Zn transporter located in the vesicles, which is the orthologue of *hZnT4*. Knocking down this gene ubiquitously induces larval and embryonic lethality, as well as the expression of MtnB [123]. This indicates that dZnT33D is an important regulator of intracellular Zn homeostasis, although it does not play a role in the dietary Zn absorption.

Zn reabsorption and excretion are also important processes in the homeostasis of this metal. dZnT63C is also localized in the basolateral membrane of cells in Malpighian tubules, which suggests its implication in reabsorption of Zn [121]. *dZnT35C* and *dZip71B* are counterparts in Zn excretion. *dZnT35C* (orthologue of *hZnT3* and *hZnT8*) is located in the apical membrane in the cells and mutant flies that has accumulated Zn, which is in support of a role in Zn excretion [124]. *dZip71B* (orthologue of *hZIP5*) is situated in the basolateral membrane, with gene knockdown producing a hypersensitivity to Zn overload similar to *dZnT35C*, although this does not happen on normal media. In contrast to *dZnT35C*, *dZip71B* knockdown shows decreased Zn content, implying that dZip71B acts upstream of dZnT35C to drive Zn excretion in the body [125]. It is suggested that dZip71B functions in transporting Zn from body into Malpighian tubules and acts together with apically localized dZnT35C to pump Zn out of the body. Meanwhile, dZnT63C is responsible for zinc reabsorption in Malpighian tubules [125]. Finally, dZnT41F is located intracellularly, and its function is not well defined, although the principal expression seems to be in the Malpighian tubules. This suggests dZnT63C may be involved in Zn homeostasis in this organ.

There are other Zn transporters that are predicted to be ZIP and ZnT orthologues in *Drosophila*, and phylogenetic analysis indicated that they are related to mammalian genes. *dZip48C*, *dZip102B* and *dZnT49B* are the orthologues of the human genes *hZIP11*, *hZIP9* and *hZnT9* respectively. Furthermore, *dZip88E* may be an orthologue of *hZIP1* and *hZIP2* or *hZIP3.* Further studies should be carried out to demonstrate their functions. 

No studies have so far addressed the question of why there are so many zinc transporters needed to regulate Zn homeostasis. One possible explanation is the vast requirement of Zn within the animal’s body compared to other metals, such as Cu or Fe. Additionally, Zn transporters might participate in the homeostasis of different metals, because it has been reported that some of them can co-transport Zn and other metals [126,127]. *dZip99C*, a member of the ZIP family, was generally believed to import zinc, but its main function is as a Fe exporter for the secretory pathway [110]. Mutation in the human orthologue of *dZip99C*, the *hZIP13* gene [110] causes the rare disease spondylocheirodysplasia Ehlers-Danlos syndrome-like [111,128], which is described in this revision. 

## 3. *Drosophila* as a Model of Human Inherited Diseases Related to Metal Homeostasis 

*Drosophila* has become a powerful model for studying human disorders caused by mutations in main components of pathways of cellular metal homeostasis. We find illustrative examples in Friedreich’s ataxia, an iron-related disorder; in Menkes and Wilson diseases caused by a deficiency and an excess of copper, respectively; and in spondylocheirodysplasia Ehlers-Danlos syndrome-like due to mutations in a member of the ZIP family of zinc transporters. In addition, *Drosophila* has shown its utility as an experimental model to explore the role of metal dyshomeostasis in different neurodegenerative diseases. 

### 3.1. Friedreich’s Ataxia 

Friedreich’s ataxia (FRDA) is a rare autosomal recessive disease with an estimated prevalence of 2–4/100,000 in populations of European origin [129]. It is a multisystemic condition affecting the central and peripheral nervous systems, the heart and other organs. FRDA is caused by a decrease in frataxin levels due to an intronic GAA triplet repeat expansion within *FXN*, the gene encoding this protein [130]. The GAA repeats can form a variety of unusual DNA structures when the GAA expansion reaches a certain size and have the potential to interfere *FXN* transcription through heterochromatic mechanisms [131].

Many studies in model organisms, such as *Saccharomyces cerevisiae*, *Mus musculus*, *D. melanogaster* and *Caenorhabditis elegans*, have contributed extensively to our current understanding of the frataxin function and the physio-pathological consequences of its deficiency [132]. Frataxin is a mitochondrial nuclear-encoded protein, which has a function that has still not been fully clarified, but it is accepted that frataxin is required for ISC biosynthesis and for cellular iron homeostasis [28]. Its deficit provokes dysfunction of ISC-containing enzymes, such as aconitase and some components of the mitochondrial respiratory chain complexes. Other hallmarks of frataxin deficiency are mitochondrial iron accumulation coupled to cytosolic iron depletion and increased susceptibility to oxidative stress [133]. In FRDA patients, excess of iron was first addressed in the myocardium and later reported in several tissues, such as the spleen, liver and cerebellum [134,135]. In FRDA models, mitochondrial iron accumulation was first described in yeast [136], before being described in mice [137] and *Drosophila* [138]. The role of iron in FRDA pathogenesis has been extensively investigated, specifically examining whether iron accumulation is either a primary event or an end-stage event, and focusing on the mechanism underlying iron-mediated toxicity. 

The RNAi methodology has been combined with the GAL4/UAS system to successfully knock down the *Drosophila frataxin homolog* (*fh*) [20]. This experimental strategy has provided tissue-specific and ubiquitous knockdown mutants in which *fh* suppression is dose dependent [139,140]. Such studies have indicated that frataxin function is vital during development and that different tissues show distinct vulnerability to the frataxin deficit. Llorens et al. [140] obtained a knockdown fly line with a systemic three-fold decrease of frataxin to parallel patient’s conditions more closely. Flies expressing the 30% residual frataxin have normal embryonic development, but show poor motor coordination and reduced lifespan in adulthood. These phenotypes are more severe under hyperoxic conditions, and aconitase activity is strongly reduced in this scenario. These results support the previous proposal that yeast frataxin can acts as an iron chaperone protecting aconitase against oxidative-mediated inactivation [141]. In addition, they are in favor of an important role of oxidative stress in the progression of FRDA. Most studies have suggested that iron accumulation becomes toxic in FRDA by promoting ROS production through the Fenton reaction [142].

Taking advantage of the valuable genetic screens methodology in *Drosophila*, Soriano et al. investigated whether genetic modification of pathways involved in metal homeostasis could improve the impairment in the motor performance of the FRDA model flies [143]. Knockdown of the iron homeostasis regulators *Irp1*-*A* and *Irp-1B* and their targets transferrin (*Tsf1* and *Tsf3*) and *Mvl* was sufficient to suppress this phenotype by reducing iron overload in the majority of cases [143]. The function of *Tsf3* has not been characterized yet, and its identification as a suppressor for FRDA phenotypes in flies provides indirect evidence of its involvement in iron metabolism in *Drosophila*. Similarly, genetic suppression of the mitochondrial iron importer mitoferrin [19] was able to counteract physiological and molecular phenotypes of frataxin loss-of-function in flies [27].

A novel ROS independent mechanism that may contribute to neurodegeneration in FRDA has recently been proposed using mutant flies with a chemically-induced missense mutation in *fh* [144]. This severe loss-of-function mutation causes an iron accumulation in adult photoreceptor neurons when it is selectively expressed in this tissue. No increase in ROS was detected, while sphingolipid synthesis was upregulated. This in turn activated the 3-phosphoinositide dependent protein kinase 1 (Pdk1) and myocyte enhancer factor-2 (Mef-2). Interestingly, blocking any step of the iron/sphingolipid/Pdk1/Mef-2 pathway suppresses neurodegeneration in mutant flies. Notably, this pathway is also activated in a mouse model with reduced Fxn levels in nervous systems and in the cardiac tissue of FRDA patients [145].

Overall, *Drosophila* models of FRDA support the proposal that iron plays a major role in FRDA physio-pathology. Although reduction in iron levels by iron chelation has been relatively successful in clinical trials [146,147], genetic or pharmacological intervention through the sphingolipid/Pdk1/Mef-2 pathway and pathways regulating iron homeostasis are new approaches to be explored in preclinical studies.

### 3.2. Menkes and Wilson Syndromes

Menkes and Wilson syndromes are Mendelian disorders of Cu metabolism caused by deficiency and excess of this metal, respectively [148]. Menkes disease is an X-linked recessive neurodegenerative disorder with reported incidences of 1 in 300,000 in European countries, 1 in 360,000 in Japan and 1 in 50,000–100,000 in Australia [149]. Patients suffer from skeletal defects, growth failure and progressive degeneration of the central nervous system and exists considerable variability in the severity of symptoms. The disease is caused by mutations in the *ATP7A* gene, which are mostly intragenic mutations or partial gene deletions [150]. The encoded protein is critical for intestinal absorption of Cu and its transport across the BBB. Therefore, in Menkes disease, Cu accumulates in the cells of the BBB and choroid plexus, making the metal unable to reach the neuronal tissue [148,151,152,153]. The decrease in Cu levels in the brain results in deficiencies in the activity of Cu-containing enzymes. 

Mutation in the copper transporter *ATP7B* gene causes Wilson disease, an autosomal recessive condition with an estimated wide prevalence of 1–9 in 100,000. Patients suffer from chronic liver disease, cirrhosis and several neurological symptoms due to a failure of copper excretion, which leads to an accumulation of the metal in the liver and brain [154].

As indicated before, *Drosophila* only has one Cu transporting P-type ATPase (named ATP7), which has a function that seems to be more closely related to that of ATP7A [51]. To study the role of ATP7, Norgate and colleagues [52] created a null mutation of the gene using imprecise excision of an inserted P-element. The mutant larvae were extremely lethargic in comparison with the controls, while their mouthparts were smaller and reduced in pigmentation. It was observed in first instar larvae that the upregulation of *Ctr1B* did not take place, even under Cu-limiting conditions. The authors suggested that Cu remained trapped in the gut cells and was not transported to the rest of the organism. As mutant larvae failed to grow and develop to the second instar, it was not possible to conduct any study in adulthood. To solve this problem, Bahadorani et al. [155] obtained conditional mutants in which silencing of *ATP7* by an RNAi construct was restricted to the gut cells. Tissue-specific suppression of *ATP7* also resulted in pre-adult mortality, but about 50% of the affected individuals survived to adulthood. Therefore, in this model, it was possible to obtain some adult survivors to study the effects of the transporter repression in adulthood. These adults showed an average lifespan that was slightly shorter than controls and a reduction in whole-body Cu content. In addition, they were sensitized to oxidative stress, probably due to a decrease in the activity of the Cu/Zn superoxide dismutase [155]. Cu supplementation and *MTF*-1 overexpression enhanced pupal survival to the adult stage, and based on this later finding, the authors suggested that induction of *MTF-1* expression could be used as an additional approach for the treatment of Menkes disease. They argued that the metallothioneins induced by MTF-1 could bind to the excess Cu accumulated in the gut, preventing its toxicity. 

A more recent work has also shown how *Drosophila* can be a powerful model to study some details of the different gene mutations responsible for a disease. Mutations in *ATB7A* and *ATP7B* are not only responsible for Menkes and Wilson diseases, but can also be involved in other disorders, such as distal motor neuropathy [156] and Alzheimer’s disease [157,158]. Mercer et al. [159] introduced different *ATP7A* and *ATP7B* pathogenic mutations into a genomic *ATP7* rescue construct. All mutations abrogated the in vivo function of *ATP7* in *Drosophila*, but the effect produced by each one of them was different. The authors also highlighted that this system can be useful to screen for compounds that would be able to restore the function of the transporters.

### 3.3. Spondylocheirodysplasia-Ehlers-Danlos Syndrome-Like 

Dyshomeostasis of Zn is related to several human disorders, such as diabetes, cancer and neurodegenerative diseases, and is also related to compromised immunity of the host. Moreover, mutations in Zn transporters are associated with hereditary human diseases. Examples of these include the transient neonatal deficiency (*hZnT2* mutations), acrodermatitis enteropathica (*hZIP4* mutations) or the spondylocheirodysplasia form of Ehlers-Danlos syndrome (*hZIP13* mutations).

The Ehlers-Danlos syndrome (EDS) is a heterogeneous group of inherited disorders of connective tissue characterized by articular hypermobility, skin hyperelasticity and tissue fragility. It affects skin, ligaments, joints, bone, blood vessels and internal organs. The natural history and mode of inheritance differ between the six major types [160]. The spondylocheirodysplasia form of Ehlers-Danlos syndrome (SCD-EDS) is a very rare condition with a worldwide prevalence of <1 in 1,000,000. It is an autosomal recessive disease caused by loss-of-function mutations in the *hZIP13* gene, which encodes a protein located in ER/Golgi apparatus [111,128]. The mouse knockout of *mZIP13* shows maturation defects affecting connective tissue development [111]. In these animals, the nuclear translocation of SMAD transcription factors is impaired, while their phosphorylation is unaffected. In addition, Zn levels in serum and isolated primary fibroblasts are not altered. At the same time, the Zn level in Golgi apparatus is upregulated and is downregulated in the nucleus, indicating an accumulation of this metal in ER/Golgi in the SCD-EDS [111]. It is proposed that zinc accumulation in ER/Golgi can compete with iron in the secretory pathway affecting the activity of enzymes in this pathway that use Fe as a cofactor. This is the case of two enzymes, namely lysyl and prolyl hydroxylases, which have a central role in the biosynthesis of collagens [161]. Mutations in the *PLOD1* gene encoding lysyl hydroxylase (LH1) cause Ehlers-Danlos type VI [162], which shares several clinical signs with SCD-EDS. In contrast to a zinc excess in SCD-EDS, a later study using human cell cultures has suggested that this disease may be the result of trapping Zn in vesicles leading to a deficiency of this metal in ER/Golgi rather than an overload and iron failing to compete with zinc in the importing assay [163].

Clarification of the SCD-EDS molecular pathology comes from analyzing the functions of *dZip99C*, the putative fly orthologue of *hZIP13*. Xiao et al. [110] generated transgenic lines of *Drosophila* to study the effects of modulating the expression of *dZip99C* in specific tissues and in the whole organism. Surprisingly, the authors found that in *Drosophila*, *dZip99C* (a presumed zinc importer) is considerably involved in dietary Fe absorption. Ubiquitous reduction of *dZip99C* expression provokes developmental arrest at the pupal stage, which can be rescued by dietary iron supplementation. Furthermore, the addition of the iron-specific chelator worsened the phenotype. Tissue-specific reduction of *dZip99C* expression in the midgut affects Fe content in whole fly, with a reduction of 50%, although the Zn level is unaltered. However, Fe levels are not reduced in the cytosol of enterocytes, which suggests that *dZip99C* is involved in Fe extrusion from the gut to the body. The authors investigated the relation of dZip99C and ferritin on iron assimilation, because *Drosophila* ferritin transport absorbed dietary iron through the secretory pathway for its systemic use [13]. Thus, dZip99C is essential for ferric iron loading in the secretory pathway. 

The intracellular location of dZip99C was examined in human intestinal Caco2 cells transfected with a myc-tagged dZip99C construct and in the fly gut. Both cases demonstrated that dZip99C is located in ER/Golgi in a similar way as its human counterpart. By means of radioactive Fe transport assay, it was also shown that dZip99C is able to transport Fe in *Escherichia coli*. The authors demonstrated that the human gene *hZIP13* but not *hZIP7*, another zinc transporter similar to *hZIP13* belonging to the same subfamily of LIV-1 transporters, rescues the developmental defects caused by knocking down *dZip99C* in flies. These results show that *dZip99C and hZIP13* are orthologues. The main function of *dZip99C* and probably for *hZIP13* is transporting iron into the secretory pathways. These results suggest that iron dyshomeostasis instead of zinc is the major cause of SCD-EDS. Mutations in *hZIP13* may compromise iron content into ER/Golgi, affecting the functions of lysyl and prolyl hydroxylases that are essential for the stability of the collagen triple helix. Further research in human tissues and other experimental models are needed to confirm iron dyshomeostasis in SCD-EDS.

### 3.4. Huntington’s Disease

Huntington’s disease (HD) is a neurodegenerative disorder characterized by movement alterations, psychiatric disturbances and cognitive dysfunction. It is transmitted in an autosomal dominant manner and its prevalence ranges from 5.96–13.7 cases per 100,000 in North America, northwestern Europe and Australia [164]. HD is caused by a CAG triplet repeat expansion located in exon 1 of the *HTT* gene, which encodes an expanded polyglutamine (polyQ) tract in the huntingtin (Htt) protein [165].

The mutant Htt-exon1 protein, carrying the polyQ tract, forms aggregates in vitro and in vivo [166]. Interestingly, it has been shown that the first 171-amino acid fragment of both normal and mutant proteins interacts with Cu [167] and that the metal favors the aggregation [168]. It is highly relevant that an increase in Cu levels has been reported in the brains of patients, as well as in mouse and rat models of HD [167,169,170].

The important role of Cu in HD has been highlighted using a *Drosophila* model of the disease [171]. Tissue-specific expression of the human Htt exon 1 protein carrying the polyQ expansion has been analyzed in flies. Expression of this mutated protein in the peripheral nervous system of the fly provokes alterations in motor coordination. These defects are ameliorated by reducing Cu import via expression of a *Ctr1B* RNAi. In contrast, these defects are exacerbated when reducing Cu export by an *ATP7* RNAi. Flies expressing the human mutant Htt in the eyes show photoreceptor degeneration, which is rescued by *Ctr1B* RNAi or *ATP7* overexpression and enhanced by *ATP7* RNAi. Analyzing the aggregation of Htt-exon1 polyQ protein in the brains of model flies, it is also observed that aggregation is alleviated by *Ctr1B* RNAi or *ATP7* overexpression and increased by *ATP7* RNAi. To further prove the key role of Cu in HD, Xiao et al. [171] mutated two critical residues potentially implicated in the binding of Cu to Htt exon1-polyQ. By doing so, the toxicity of the mutant protein was drastically reduced, and the subsequent phenotypes seemed to be no longer dependent on Cu. Based on these results, it was shown that HD involves both polyQ toxicity and a Cu-modulating effect, with these two pathways needing consideration in the treatment of the disease [171].

### 3.5. Parkinson’s Disease

Parkinson’s disease (PD) is a common neurodegenerative disorder characterized by movement impairments and mood alterations. It is caused by loss of dopaminergic (DAergic) neurons in the substantia nigra pars compacta (SNpc) [172], which is mostly due to a sporadic origin with a contribution of both environmental and genetic factors. There is also a small percentage of familial cases caused by single gene mutations that can be modeled in organisms like *Drosophila*. For instance, mutations in *PARK2* are responsible for a form of juvenile Parkinsonism [173]. *PARK2* codifies for PARKIN, an E3 ubiquitin ligase [174,175]. It has been observed that flies lacking the orthologue gene *parkin* show mitochondrial abnormalities in muscle and germline tissues in addition to increased sensitivity to oxidative stress [176,177,178]. Saini et al. [179] reported interactions of Fe and Cu with the parkin deficiency in *Drosophila.* Flies carrying a null mutation (*park^25/25^*) of *parkin* [177] show a reduction in lifespan, which is rescued using the Cu chelator bathocuproine disulfonate or the Fe chelator bathophenanthroline sulfonated sodium salt. These flies also show an ROS reduction in their heads after the chelator treatments. The authors argued that the lifespan extension might be due to oxidative stress reduction through the sequestration of redox-active Cu and Fe. They further prove the interaction between parkin and Cu by overexpressing *Ctr1B* in the fly eye using the *GMR* promoter. An increased amount of imported Cu through Ctr1B along with the parkin deficiency was enough to induce a rough eye phenotype. This phenotype was not present in *park^25/+^* flies overexpressing the Cu transporter. Using the *parkin*-deficient flies, it was also shown that other metals could also influence the severity of PD. *Parkin* mutant flies showed an increase in their survival and eclosion frequency when they were fed on a high-zinc food [180]. These effects could be due to the antioxidant properties that Zn may exert by being able to protect protein sulfhydryl groups, compete against redox-active metals or upregulate the expression of metallothioneins [180,181].

Previous data have already shown that several metals could damage dopaminergic neurons in humans and other model organisms, leading to movement alterations [182,183,184,185,186,187]. This effect was also further explored in *Drosophila* by treating wild type flies (Canton-S) with iron, copper and manganese [6]. An acute and chronic exposure to high doses of these metals clearly reduced the lifespan of the flies and the number of neurons of dopaminergic neuron clusters in their brains.

One of the main hallmarks of PD is the presence of Lewy bodies, which are mainly composed of protein α-synuclein [188]. The toxicity of α-synuclein in dopaminergic neurons may be related to the formation of protein oligomers. It has been observed that some mutations of the α-synuclein gene (*SNCA*), such as A53T and A30P, provoke a faster aggregation in the mutant protein compared to the wild type one. Moreover, it has been reported that iron binding can increase α-synuclein aggregation [189] and that the mutant proteins may have a higher ferrireductase activity, converting Fe^3+^ to the more reactive Fe^2+^ [190]. This interaction between α-synuclein and Fe has also been recently explored in *Drosophila*. Flies expressing mutant A53T or A30P α-synuclein in dopaminergic neurons show a higher decline in motor function when treated with Fe compared to flies expressing the wild type protein in the same Fe conditions [191]. Similarly, Fe only induces a significantly selective dopaminergic neuron loss in the PPM3 neuron cluster only in the flies expressing the mutant proteins.

### 3.6. Alzheimer’s Disease

Alzheimer’s disease (AD) is a progressive neurodegenerative condition, which is the main cause of dementia among aged people [192]. The disorder is characterized by the shrinkage of several regions in the hippocampus and cortex implicated in memory and cognition. Characteristically, there is an accumulation of amyloid plaques rich in the β-amyloid (Aβ) peptide and neurofibrillary tangles with hyperphosphorylated tau in the brain [193]. The toxicity of Aβ aggregates may be due to degradation of the barrier properties of cellular membranes [194,195], binding to cell receptors [196,197,198,199] and the generation of oxidative stress, since Aβ aggregates can form complexes with redox active metals, such as Fe and Cu [200,201]. *Drosophila* has also been used to study the toxicity of the Aβ peptide and its interaction with metals. Expressing the 42-amino acid isoform of Aβ (Aβ42) in the neurons of flies resulted in a decrease in lifespan and locomotor performance in comparison with flies expressing the less toxic 40-amino-acid isoform of Aβ (Aβ40) [202]. The Aβ40 isoform does not contain the final two hydrophobic residues that make the Aβ42 isoform more prone to aggregation. Expression of the Aβ42 carrying the Arctic mutation (E22G) [203], which further increases aggregation, produces even more severe phenotypes. By means of microarray analysis and genetic screen, it was shown that oxidative stress plays an important role in the toxicity of Aβ42. In fact, the levels of carbonyl groups in the protein extracts of fly heads increase with the expression of the wild type Aβ42 or the Arctic Aβ42, which indicates the presence of oxidative damage. In the same study, a strong interaction between Fe and the toxicity of the Arctic Aβ42 is pointed out. The expression of the heavy or light subunits of ferritin rescues the phenotypes associated with the Aβ42 toxicity. After this rescue, flies have an increased survival and motor performance, while carbonyl groups’ content is reduced. It was suggested that the effect of the ferritin subunits could be due, at least in part, to a reduction in the hydroxyl radical production by sequestering the Fe ions. In a further study, the same group confirmed the relevance of Fe in the Aβ toxicity [204]. Flies treated with the Fe-specific chelator YM-F24 had a reduced toxicity of this peptide, while knocking down the endogenous ferritin worsened it. Moreover, Fe actively affects the aggregation process of Aβ, delaying the formation of well-ordered aggregates and possibly promoting its toxicity by this effect. In a recent study, it was shown that Fe affects the dimerization of Aβ through the interaction with three N-terminal histidines in the peptide [205]. To analyze their position-dependent and position independent effects, histidines were systematically substituted by alanine residues in Aβ. It was found that the susceptibility of the flies to oxidative stress was determined only by the number of histidines.

Most AD clinical forms have sporadic etiology, but there are also some familial forms linked to mutations in the *APP* (amyloid precursor protein), *PSEN1* (presenilin 1) and *PSEN2* (presenilin 2) genes [206]. Presenilins act as catalytic subunits of the γ-secretase multi-protein complex [207], which in turn is involved in the cleavage of the amyloid precursor protein. Presenilins also seem to have a role in Cu and Zn uptake in mammalian systems [208]. This novel role of presenilins has been studied in *Drosophila* by tissue-specific knocking down of the single presenilins orthologue (*PSN*) in the digestive system [209]. It was observed that the *PSN* knockdown flies had an increased Cu tolerance due to a reduced Cu uptake. These flies showed a reduction in total Cu levels and were less sensitive to excess dietary Cu when overexpressing *ATP7*. Taking into account that presenilins are involved in the trafficking and localization of several proteins [210,211], it was suggested that an impaired cellular localization of Ctr1A and Ctr1B could be responsible for reduced Cu uptake.

## 4. *Drosophila* as Model for Testing Metal Toxicity

Heavy metals are natural components of the Earth’s crust. Contamination of heavy metals in the environment is a major global concern, because of toxicity and threat to human life and ecosystems. In humans, exposure to heavy metals is mostly occupational [212]. Metal-containing dusts and fumes are generated along the complete life cycle of metal objects from ore mining through smelting and final product manufacturing to waste management and recycling. These dusts and fumes are found in the workplace atmosphere, sometimes at hazardous concentrations. Many metals are xenobiotics because they used to have a minimal presence (and hence, bioavailability) before emission into the environment from industry. Furthermore, they either are completely useless and toxic for the human organism (e.g., mercury, lead or cadmium) or are essential micronutrients, but toxic when given in excess concentrations (e.g., iron and copper). The toxic effects of heavy metals and their consequences show a broad spectrum [213]. For example, metals, such as arsenic, cadmium, lead and mercury, cause many altered conditions including hypophosphatemia, heart disease, liver damage, cancer, central nervous system injury and sensory disturbances.

Based on the chemical and physical properties of heavy metals, three major molecular mechanisms of toxicity have been distinguished [214]. The first is production of ROS by auto-oxidation and the Fenton reaction, which is characteristic for transition metals, such as iron or copper. The second is blocking of essential functional groups in biomolecules. These reactions have mainly been reported for non-redox-reactive heavy metals, such as cadmium and mercury. The final mechanism involves displacement of essential metal ions from biomolecules, which occurs with different types of heavy metals. As a result of these toxic effects, various cellular metabolic processes are inhibited, which are detrimental to the cell. *Drosophila* has also contributed to our understanding of the underlying mechanisms associated with metal toxicity. Alterations on the cell cycle, circadian rhythms and enzymatic pathways, DNA repair impairment in addition to genotoxicity are several consequences of metal toxicity found in flies. Table 4 summarizes the main sources of metal contamination and human exposition, as well as the clinical symptoms that have been reported in humans and *Drosophila* findings.

## 5. Conclusions 

As a model organism, *Drosophila melanogaster* has been intensely studied for over a century. Today, the knowledge related to the biology of the fly and the tools to work with it vastly exceed those available for many other model organisms. The conservation of many cellular and organismal processes between humans and flies has also made *Drosophila* one of the best choices to start studying many issues relevant for human health. The fly contributes greatly to achieving a better understanding about metal uptake, distribution, storage or excretion, as well as in establishing the underlying toxicity mechanisms of metals and their role in the pathophysiology of many human diseases. We have presented here some illustrative examples of how *Drosophila* can be used to model and study rare diseases in which metals are the main players; other highly prevalent neurodegenerative disorders in which the importance of metals is increasingly being considered; or the toxicity of high levels of several metals on their own. Nevertheless, there are still many other metal-related disorders and questions that can be addressed by taking advantage of *Drosophila*, evidencing the great potential of this little organism.

## Figures and Tables

**Table 1:** Conserved proteins in iron homeostasis.

Human Gene	Primary Metals	Metal-Related Function	*Drosophila* Orthologue	References
*Divalent Metal Transporter 1* (*DMT1*, *SLC11A2*)	Fe	Divalent metals transport	*Malvolio* (*Mvl*)	[10,11]
		Iron absorption		
*Ferritin Heavy Chain 1* (*FTH1*)	Fe	A component of ferritin	*Ferritin 1 heavy chain homologue* (*Fer1HCH*)	[12,15]
		Iron storage		
*Ferritin Light Chain* (*FTL*)	Fe	A component of ferritin	*Ferritin 2 light chain homologue* (*Fer2LCH*)	[12,15]
		Iron storage		
*Ferritin Mitochondrial* (*FTMT*)	Fe	Iron storage	*Ferritin 3 heavy chain homologue* (*Fer3HCH*)	[19]
		Oxidative stress protection		
*Transferrin* (*TF*)	Fe	Serum iron binding transport protein	*Transferrin 1* (*Tsf1*)	[13,14]
*Aconitase 1* (*ACO1*)	Fe	Iron sensor	*Iron regulatory protein 1A* (*Irp-1A*)	[16,18]
			*Iron regulatory protein 1B* (*Irp-1B*)	
*Mitoferrin 1* (*SLC25A37*)	Fe	Mitochondrial iron importer	*Mitoferrin* (*mfrn*)	[19]
*Frataxin* (*FXN*)	Fe	Mitochondrial iron chaperone	*Frataxin* (*fh*)	[20]
*Duodenal cytochrome b* (DCYTB, *CYBRD1*)	Fe	Ferric-chelate reductase that reduces Fe^3+^ to Fe^2+^	*No extended memory* (*nemy*)	[21]
			*CG1275*	
*Hephaestin* (*HEPH*)	Fe	Ferroxidase activity oxidizing Fe^2+^ to Fe^3+^	*Multicopper oxidase-1* (*Mco1*)	[22]
*Ceruloplasmin* (*CP*)	Fe	Ferroxidase activity oxidizing Fe^2+^ to Fe^3+^	*Multicopper oxidase-3* (*MCO3*)	[23]

Gene symbols for human genes are indicated according to the Human Genome Organization Gene Nomenclature Committee (HGNC) (Available online: http://www.genenames.org). An alias is indicated in several cases. *Drosophila* gene symbols are cited in agreement with the Flybase (Available online: http://flybase.org).

**Table 2:** Conserved proteins in copper homeostasis.

Human Gene	Primary Metals	Metal-Related Function	*Drosophila* Orthologue	References
*Solute carrier family 31 member 1* (*SLC31A1, CTR1*)	Cu	Copper uptake	*Copper transporter 1A* (*Ctr1A*) *Copper transporter 1B* (*Ctr1B*) *Copper transporter 1C* (*Ctr1C*)	[33,41,42]
*Copper chaperone for superoxide dismutase* (*CCS*)	Cu	Chaperone; copper donor to SOD1	*Copper chaperone for superoxide dismutase* (*Ccs*)	[44,45]
*Cytochrome c oxidase copper chaperone* (*COX17*)	Cu	Chaperone; copper donor to COX11 and SCO1	CG9065	[46]
*Cytochrome c oxidase copper chaperone* (COX11)	Cu	Chaperone; copper transfer to cytochrome c oxidase	CG31648	[46]
*Cytochrome c oxidase assembly protein* (SCO1)	Cu	Chaperone; copper transfer to cytochrome c oxidase	*Synthesis of cytochrome c oxidase* (*Scox*)	[46,47]
*Antioxidant 1 copper chaperone* (*ATOX1*)	Cu	Chaperone; copper donor to ATP7A and ATP7B	*Antioxidant 1 copper chaperone* (*Atox1*)	[48]
*ATPase copper transporting α* (*ATP7A*)	Cu	Copper delivery to proteins in the secretory pathway; copper efflux	*ATP7* (*ATP7*)	[49,50,51,52,53,54,55]
*ATPase copper transporting β* (*ATP7B*)	Cu	Copper delivery to proteins in the secretory pathway; copper efflux	*ATP7* (ATP7)	[49,50,51,52,53,54,55,56]

Gene symbols for human genes are indicated according to the Human Genome Organization Gene Nomenclature Committee (HGNC) (Available online: http://www.genenames.org). An alias is indicated in several cases. *Drosophila* gene symbols are cited in agreement with the Flybase (Available online: http://flybase.org).

**Table 3:** Conserved proteins in zinc homeostasis.

Human Gene	Primary Metals	Metal-Related Function	*Drosophila* Orthologue	References
*SLC30A1* (*hZnT1*)	Zn	Exporting cytosolic zinc into the extracellular space	*dZnT63C* *dZnT77C*	[80]
*SLC30A10* (*hZnT10*)	Mn, Zn	Zinc transporter localized to early/recycling endosomes or Golgi		[81,82]
*SLC30A2* (*hZnT2*)	Zn	Transporting zinc into the lumen of vesicular compartments	*dZnT33D* *dZnT35C*	[83,84,85]
*SLC30A3* (*hZnT3*)				[86]
*SLC30A8* (*hZnT8*)				[87,88]
*SLC30A4* (*hZnT4*)	Zn	Maintenance of cytosolic zinc; homeostasis by controlling zinc; translocation to the lysosomes	*dZnT41F*	[89]
*SLC30A7* (*hZnT7*)	Zn	Transports zinc into early secretory pathway and contributing to its homeostatic control	*dZnT86D*	[90,91]
*SLC30A9* (*hZnT9*)	Zn	No zinc transport functions; acts as nuclear receptor coactivator	*dZnT49B*	[92]
*SLC39A1* (*hZIP1*)	Zn	Imports zinc from the extracellular space	*dZip42C.1* *dZip42C.2* *dZip89B* *dZip88E*	[93,94]
*SLC39A2* (*hZIP2*)				[95,96]
*SLC39A3* (*hZIP3*)				[97,98]
*SLC39A5* (*hZIP5*)	Zn	Zinc importer	*dZip71B*	[99,100]
*SLC39A6* (*hZIP6*)	Zn	Zinc importer that can be a growth factor-elicited signaling molecule	*fear of intimacy* (*foi*)	[101,102]
*SLC39A10* (*hZIP10*)				[103,104]
*SLC39A7* (*hZIP7*)	Zn	Zinc importer from endoplasmic reticulum and Golgi apparatus; implicated in the glycemic control in skeletal muscle	*Catecholamines* up (*catsup*)	[105,106]
*SLC39A9* (*hZIP9*)	Zn	Zinc importer localized to the Golgi apparatus and the cell surface; plays a crucial role in B-cell receptor	*dZip102B*	[107,108]
*SLC39A11* (*hZIP11*)	Zn	Not well defined	*dZip48C*	[109]
*SLC39A13* (*hZIP13*)	Fe	Mobilizes zinc from the lumen of Golgi apparatus and cytoplasmic vesicles to cytosol and plays a pivotal role in cellular signaling	*dZip99C*	[110]
	Zn			[111]

Gene symbols for human genes are indicated according to the Human Genome Organization Gene Nomenclature Committee (HGNC) (Available online: http://www.genenames.org). An alias is also indicated for them. *Drosophila* gene symbols are cited in agreement with the Flybase (Available online: http://flybase.org).

**Table 4:** Main sources, routes of human exposure, symptoms and contributions of research in *Drosophila*.

Metal	Natural and Human Sources	Main Human Exposure	Symptoms	*Drosophila* Findings	Reference
Aluminum	Water treatment agents, aerosol, cosmetics, food additives, beverage cans, cookware, fireworks, explosives, rubber manufacturing	Drinking water, food, inhalation, dermal contact, pharmaceuticals	Mouth ulcers, skin lesions, bone, lung and brain damage, neurodegeneration, loss of memory, problems with balance and loss of coordination	Neurological injury, neurodegeneration, developmental alterations, behavior impairment, lifespan reduction and daily rhythm alterations, increase iron accumulation and ROS production	[215,216,217,218,219,220]
Arsenic	Arsenic minerals, sedimentary bed rocks, mining, melting, pesticides, fertilizers, drugs, soaps	Drinking contaminated water	Abnormal heart beat, damage in blood vessels, skin lesions, cancer, neurological problems, high rate of mortality	Genotoxicity of methylated metabolites, susceptibility related with genes of the biosynthesis of glutathione, brain injury, developmental alterations	[215,221,222,223,224,225,226]
Cadmium	Batteries, plastics, pigments, weathering, volcanic eruptions, river transport, fertilizers, pesticides, smelting, mining	Contaminated food and drinking water, inhalation, occupational exposure	Renal dysfunction, bone and lung damage and kidney disease	Changes in transferase enzymatic activity, stress response, cell cycle alterations, interference in DNA repair mechanism	[215,227,228,229,230]
Chromium	Burning of petroleum, coil and oil, pigment oxidants, fertilizers, metal planting tanneries, sewage, metallurgy, paper production	Water, occupational exposure	Ulcers, fever, renal failure, liver damage and hemorrhagic diathesis	DNA damage, alterations in pre- and post-replication mechanism implicated in repair DNA, changes in humoral innate immune response	[215,227,231,232]
Lead	Pipes, paints, gasoline, cosmetics, bullets, pesticides, fertilizers, mining, fossil fuel burning	Occupational exposure, food, smoking and water	Arthritis, renal dysfunction, vertigo, hallucinations, birth defects, mental retardation, psychosis, hyperactivity, autism, brain damage	Alterations in presynaptic calcium regulation, identification of QTL associated with behavioral lead-dependent changes, weak mutagenic effect, endocrine disruption	[215,227,233,234,235,236,237,238,239,240]
Manganese	Steel industry, mining, soil erosion, fungicides, fertilizers, dry-cell batteries, fireworks, ceramics, paint, cosmetics	Occupational exposure, water and food	Manganism, tremors, psychosis, fatigue, irritability	Reduced cell viability, induction of ROS, decrease in lifespan and locomotor activity	[6,216,241,242,243,244,245,246]
Mercury	Agriculture, mining, wastewater discharges, batteries	Contaminated water and marine food	Brain damage, memory problems, depression, hair loss, fatigue, tremors, changes in vision and hearing	Morphometric changes, interference in cellular signaling pathways and enzymatic mechanisms, inhibition of Notch cleavage by γ-secretase	[6,215,243,244,245,246,247,248,249]

## Abbreviations

ACO	Aconitase
AD	Alzheimer’s disease
Aβ	β-amyloid peptide
BBB	Blood brain barrier
CCS	Copper chaperones for superoxide dismutase
CP	Ceruloplasmin
CTR1	Copper Transporter-1
Cu	Copper
DAergic	Dopaminergic
DMT1	Divalent metal transporter-1
EDS	Ehlers-Danlos syndrome
ER	Endoplasmic reticulum
Fe	Iron
FRDA	Friedreich’s ataxia
FXN	Frataxin
HD	Huntington’s disease
Htt	Huntingtin
IRE	Iron-responsive element
IRP	Iron regulatory protein
ISC	Iron-sulfur cluster
LH1	Lysyl hydroxylase
MCO	Multicopper oxidase
Mef-2	Myocyte enhancer factor-2
MRE	Metal response elements
MTF-1	Metal-responsive transcription factor-1
PD	Parkinson’s disease
Pdk1	Protein kinase-1
polyQ	Polyglutamine
QTL	Quantitative trait locus
RNAi	RNA interference
ROS	Reactive oxygen species
SCD-EDS	Spondylocheirodysplasia form of Ehlers-Danlos syndrome
SNpc	Substantia nigra pars compacta
SOD	Superoxide dismutase
TF	Transferrin
Zn	Zinc
